# Everybody is able to reflect, or aren't they? Evaluating the development of medical professionalism via a longitudinal portfolio mentoring program from a student perspective

**DOI:** 10.3205/zma001533

**Published:** 2022-02-15

**Authors:** Sylvia Schrempf, Lene Herrigel, Justus Pohlmann, Jan Griewatz, Maria Lammerding-Köppel

**Affiliations:** 1 Competence Centre for University Teaching in Medicine Baden-Württemberg, Tübingen, Germany; 2 University of Tübingen, Faculty of Medicine, Student Council, Tübingen, Germany

**Keywords:** professional reflection, portfolio, mentoring, medical education, competency orientation, hidden curriculum

## Abstract

**Introduction: **Reflective competence is fundamental for responsible medical practice and must be systematically incorporated in medical training. To promote this, a longitudinal portfolio-based mentoring program was made mandatory at the Medical Faculty of the University of Tübingen in 2013. This study examines medical students' attitudes toward professional reflection and toward the program in general to draw conclusions about conditions as well as the needs-based design of the program.

**Method: **In winter semester 2017/18, a retrospective questionnaire survey with free text fields was conducted (total sample: N=1.405; students S 1-9; response 37%; S 1-4 “Pre-clinic”: n=231; S 5-9 “Clinic”: n=241). Opinion trends of semester groups were identified through seven semi-structured interviews with semester speaker and peer tutors.

**Results: **Differences in understanding and attitudes resulted in three positions: 1=approval, 2=ambivalence, 3=rejection. All three groups included individuals from pre-clinical and clinical settings with varying levels of experience. Prior experience and hidden curriculum influenced the position. Opinion trends confirmed the feedback.

**Conclusion:** Although reflection appears in the National Competence-based Learning Objectives Catalogue for Medicine (NKLM), reflective competence is not regarded as equivalent to other study content. Motivation, commitment on the part of the mentors, and a trusting mentor-mentee relationship are effective. The flexibility of the portfolio in terms of content and methodology, as well as the curricular integration of the program are also beneficial.

## Zusammenfassung

**Einleitung:** Reflexionskompetenz ist eine Grundlage verantwortungsvollen ärztlichen Handelns und muss in der ärztlichen Ausbildung systematisch adressiert werden. Zur Förderung dieser wurde 2013 an der Medizinischen Fakultät der Universität Tübingen ein longitudinales Portfolio-gestütztes Mentoring-Programm verpflichtend eingeführt. Diese Studie untersucht die Einstellungen der Medizinstudierenden auf die professionelle Reflexion und das Programm im Allgemeinen, um Rückschlüsse auf Bedingungen und bedürfnisgerechte Gestaltung des Programms zu ziehen.

**Methode: **Im WS 2017/18 wurde retrospektiv eine Fragebogenbefragung mit Freitextfeldern durchgeführt (Gesamtstichprobe: N=1.405; Studierende FS 1-9; Rücklauf 37%; FS 1-4 „Vorklinik“: n=231; FS 5-9 „Klinik“: n=241). Meinungstrends der Semestergruppen wurden über 7 semi-strukturierte Interviews mit Semestersprecher*innen und Peer-Tutor*innen ermittelt.

**Ergebnisse: **Die Unterschiede in Verständnis und Einstellungen ergaben 3 Positionen: 1=Zustimmung, 2=Ambivalenz, 3=Ablehnung. In allen 3 Gruppen waren Personen aus Vorklinik und Klinik mit unterschiedlichem Erfahrungsgrad. Vorerfahrung und hidden curriculum nahmen Einfluss auf die Position. Die Meinungstrends bestätigten das Feedback.

**Schlussfolgerung: **Obgleich im nationalen kompetenzbasierten Lernzielkatalog Medizin (NKLM) verankert, wird die Reflexionskompetenz noch nicht gleichwertig zu anderen Studieninhalten gesehen. Erfolgswirksam sind Motivation, Engagement der Mentor*innen und ein vertrauensvolles Mentor-Mentee-Verhältnis. Förderlich sind zudem eine inhaltliche und methodische Flexibilität des Portfolios sowie die curriculare Anbindung des Programms.

## Introduction

Professional reflection holds great importance for patient safety and lifelong learning in the medical profession [1], [2], [3]. The ability to self-reflect is internationally ranked among the core clinical competencies to be learned in medical school [4], [5], [6], [7], [8]. It is a complex, iterative cognitive process that is not inherently comprehended and mastered by students [9], [10]. Also, the fact that aspects of professionalism, such as altruism, empathy, and conscientiousness may diminish during medical school demonstrates the relevance of preventive approaches [11], [12], [13]. That is why professional reflection should already be promoted at the beginning of a medical degree with instructions, support, and feedback [14], [15]. In the traditionally science-oriented German medical curricula, reflection as a key competence has thus far been addressed implicitly, whereas it has been addressed explicitly in target projects in various (pre) clinical courses [16], [17]. Since 2015, the German national competence-based learning objectives catalogue in medicine [http://www.nklm.de] has demanded professional self-reflection to be systematically integrated into medical curricula as an essential competence of the medical field. Given that in addition to professional competence, aspects such as reflective competence and explicit professional development must be addressed in a specific manner, curricula need to be differently emphasized and re-aligned [18].

Despite the widely recognized importance of reflective competence and the demand for its purposeful incorporation, reactions regarding its teaching among students and teachers vary from enthusiastic approval to rigorous rejection [8], [19]. Comprehension, value, and attitude as well as professional socialization influence the views on professional reflection and related curricular measures [5]. For years, there have been controversial debates about whether and, if so, how reflection processes can be mediated, externally controlled, and evaluated [20]. This is aggravated by the fact that an accepted uniform definition of “reflection” has not yet been agreed upon, which often leads to misunderstandings [8]. Nguyen et al [21] developed a helpful model of reflection consisting of five core components: “Reflection is defined as the process of engaging the self in attentive, critical, exploratory and iterative interactions with one’s thoughts and actions, and their underlying conceptual frame, with a view to changing them and a view on the change itself.” They added two relevant extrinsic factors: triggers (e.g., real experiences) and context. The differentiation of reflection from other thought processes is thereby possible [21].

There is a high need for appropriate teaching methods and tools [2], [8], [22]. Ugur et al. [8] stated that there is a great heterogeneity in the way reflection is taught to medical students. According to their meta-analysis, guidance on reflective writing and feedback seem to improve students' reflective competences the most. Portfolio formats combined with mentoring are common in education and practical training worldwide, although with mixed success [5], [19], [23], [24].

In the environment outlined above, the Medical Faculty of Tübingen decided to develop an obligatory portfolio-based mentoring program (Studienpat*innen-Lernportfolio-Programm, SLP). The aim was to promote students' reflective competence during their studies through interconnecting methodological elements and formative feedback, but without summative grading. In the winter semester of 2017/18, after five years, a total of almost 1.800 students from 10 semesters were supervised by 220 mentors. This was a promising position to start from for a retrospective cross-sectional survey. 

The objective of this study is to examine the attitudes of medical students while learning to reflect through the SLP and to identify aspects of perception and its effect. Qualitative feedback from students is then used to draw conclusions about conditions and needs-oriented design for a longitudinal program like this. The guiding questions are:

- How do students evaluate professional reflection and the SLP?
- Which aspects influence the perception and impact of the SLP along with its components?
- What possibilities for improvement do they see?

## Method

### Program description

**Figure 1:**
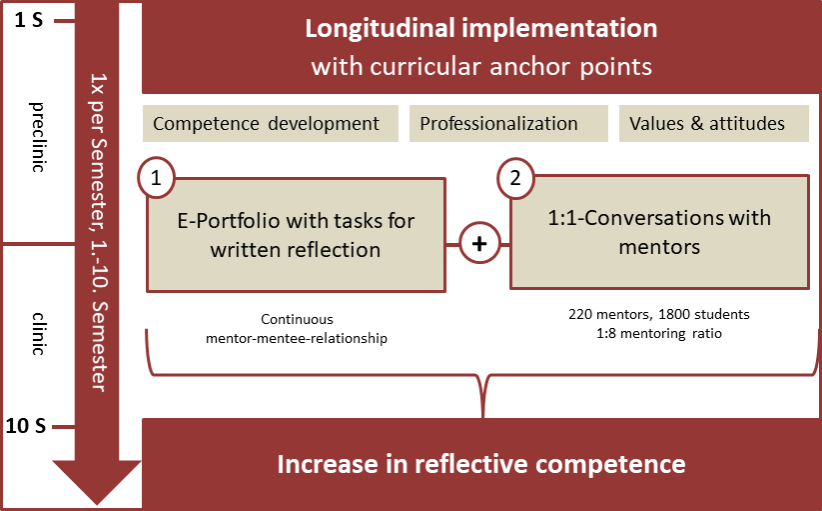
Schematic overview of the constituent elements of the Tübingen SLP-Programm.

Since 2013, the SLP has been established at the Medical Faculty of Tübingen. Each semester, new students are admitted to the program, which they are required to complete by their 10^th^ semester. The concept and flow of the SLP program are shown schematically in figure 1 (Fig. 1). Two central components determine the program:

1. **Reflective e-portfolio** (via learning management system ILIAS) with written reflection and self-assessment of professional competence development relevant to medicine (Reflection: ALACT [25]). The portfolio contains a “slot” for each competency, which is filled with reflection texts, action plans or other evidence for the learning focus of a semester. Over time, the acquisition of competencies is repeatedly highlighted in the context of several subjects and situations (developmental spiral). Optionally, a learning-relevant reflection topic can be freely chosen (individualization, flexibility).
2. **Conversations with the mentor.** A mentor usually supervises 8 students. In a 1:1 discussion (at least once per semester), the mentor questions the submitted reflection, gives formative feedback and advises if necessary (no evaluation of the content or grading of the reflection). The logical sequence of the reflection steps and diligence are crucial. The mentors are teaching doctors or scientists from disciplines related to medicine (80:20; recruited on recommendation by students, mentors, Competence Center for University Teaching in Medicine, Office of the Dean of Students). They are trained in a workshop and semi-annual updates. The mentor-mentee relationship remains predominantly (>80%) from the 1st to the 10^th^ semester (change of mentor e.g. in case of job change, family break, irreconcilable differences).

#### Study design and research methods 

A cross-sectional survey was conducted retrospectively in the winter semester 2017/18:

1. questionnaire survey through structured online surveys (with free text fields),
2. semi-structured interviews for content validation and deepening.

#### Online-survey

##### Instrument

The online questionnaire (SoSci Survey) collected demographic data and asked (based on 16 question categories and 24 items, using a five-point Likert scale) about the program and framework conditions, (written) reflection, and mentoring (meaning, profit, suggestions for improvement). The thematic blocks contained statements for evaluation and explanation in free text fields.

##### Study participants

All students in semesters (S) 1-9 who had agreed to participate anonymously in the study received the link by mail (N=1.476). Students critical of guided reflection were explicitly invited to participate in order to get to know their views better and to explicitly include them. S 10 was not surveyed due to upcoming final exams. 71 mails could not be delivered (total sample: N=1.405).

##### Evaluation

The first number of a student's code indicates the S and the next the input number of the questionnaire (example from S 3: B3-52). Only the free texts were used for the evaluation. The data material was imported into MAXQDA for structured qualitative content analysis [26]. For this purpose, a coding guideline was determined in advance and a category system was developed. The main categories were formed deductively, subcategories were derived inductively from the material. Further subcategories were assigned to the subcategories, so that in the end 21 categories were formed. The free text comments were evaluated independently by two persons. In a discursive consensus finding process, disagreements were resolved: e.g., further categories were formed as well as definitions, coding notes, keywords, or anchor examples were added to the previous categories. The text material was reviewed again at a later stage by two additional persons with the help of the coding guide. 

#### Semi-structured Interviews 

##### Participants

7 semester speakers (elected representatives of the students) and peer tutors with at least 1 year of experience were asked to report opinion trends and moods from their semester group (preclinical n=3, clinical n=4; basis: experience from higher-level function, own evaluations; audio documentation).

##### Instrument

The content of the guide was based on the online survey and literature. Interview transcripts documented special features, anomalies or interruptions.

##### Evaluation

Audio recordings were transcribed in MAXQDA, categorized, and analyzed as above [26]. The results were reviewed and discussed for agreements, contradictions, and new content.

## Results

### Characterization of the study participants

The response rate was moderate at 37% (n=526). After adjustment for missing data or dropout, 472 questionnaires could be evaluated (S 1-4 “Preclinical”: n=231; S 5-9 “Clinical”: n=241). Table 1 (Tab. 1) shows the sociodemographic data of the study participants. These are equally distributed across the semesters (M=58; max. 77; min. 40). 

#### Subjective attitudes toward reflection and the SLP 

Three statement categories with differences in understanding and attitudes emerged in the study population. Their positions ranged from

1. approval, to
2. ambivalence, to
3. rejection,

each with smooth transitions. Group assignment was based on individual statements. Thus, different statements from individual students could be assigned to several groups. In all 3 groups, statements from preclinical and clinical students with different levels of experience were represented. Educational level and prior experience influenced the position. Many made constructive suggestions about the program (see table 2 (Tab. 2)). Regardless of their attitude, 73% of students reported benefiting from the SLP. The following quotes representatively reflect the tenor of the statements.

#### Approving position

This group considered reflection to be fundamentally important and regularly required in their studies (B3-52; B9-18; 36.1% - 226 comments; 100 preclinical, 126 clinical). According to their own statements, some students with (nursing) training had maintained their positive *reflective behavior and didn’t change it since the beginning of their studies* (B7-38). They valued written reflection (*in-depth reflection, basis for conversation* B8-16). For some, the 1:1 *conversation always marked the end of a semester* (B3-15). Depending on their level of training and previous experience, they benefited from different aspects:

The reflection tasks and guiding questions were stimulating impulses for reflection (B1-19) and a good way to identify their way of learning (B2-23), the state of their competencies, and strengths and weaknesses (B8-11). Some would not have taken the time to do so without the program (B1-7). Preclinical and clinical students demonstrated different content interests (see table 3 (Tab. 3)). Several participants saw it as an advantage to be able to track their development over time and to be aware of their own progress (B2-50, B7-32).

Mentors had an important learner-oriented leadership role in helping students learn to reflect (B3-36), providing new perspectives and motivation (B3-52, B5-43), or helping uncertain students to realistically assess and value their own performance (B6-32). Clinical students also appreciated mentors for good personal contacts (B5-31), valuable tips, advice, and letters of recommendation (B9-54), or for easily organized job shadowing in the clinic or laboratory (B5-16). It gave them additional security to have a contact person (B2-55, B7-3). Overall, this group did well with the SLP. They suggested more formal flexibility as well as more frequent review of competence development.

#### Ambivalent “yes, but” position

Students with ambivalent attitudes found themselves in a dichotomy (27,2% - 170 comments; 71 preclinical, 99 clinical): On the one hand, they valued reflection and program (*good idea, sensible and useful, helpful* B9-29, B8-43), but on the other hand, they saw competition with other demands (*Studying already challenges me enough!* B6-50). In the critical balance, *effort and benefit* *were too unequal* (B7-12). The time factor played a major role; in particular, written reflection was perceived as additional stress during high learning pressure and exams and led to avoidance strategies (Problems: reflective writing, nature of evidence, scope, constant formulation of goals 1-64, B5-39, B8-49).

Some were looking for graduated relief: the reflection process should be ongoing, including with a mentor for each student, but not necessarily in writing (B3-20, B5-31, B9-29). Some with prior experience recommended making the SLP mandatory more for younger students straight out of school (B2-37, B6-36) and for those who had problems with their studies, e.g., organization and motivation (B3-55). These students were thus closer to the rejectionist position.

#### Rejecting position

This group was fundamentally and, in some cases, emotionally opposed to curricular promotion of reflective skills (36.7% - 230 comments; 107 preclinical, 123 clinical). The list of their “beliefs” (see table 4 (Tab. 4)) shows their convictions and solidified negative attitudes. The free texts often revealed misunderstandings, insufficient knowledge, and uncertainties. They argued with formal, methodological and ethical-legal objections: Reflection is *something very private and personal* (B1-16), which they do more honestly and authentically with themselves (B5-36) or with trusted people (B3-55), but *not under review of the university* (B5-45). They indignantly asked about legality, data protection and ethics (*It should be my own decision whether and how I reflect* B6-45). A few doubted *whether this activity [as a mentor] is comparable to a teaching position* (B5-41). Overall, this group tended to think that teaching reflective skills did not belong in medical school, especially written reflection.

#### Change of attitude

In addition, the attitude of the students could change in the course of the program. In retrospect, students perceived developments both from the negative to the positive and vice versa *(In the beginning, it was just work for me (a lot of writing), but as the semesters went by, I partly understood the meaning behind it B4-32 vs. In the beginning [...] it was good to record things in writing. Now [...] [I] don't really need that anymore* B6-39). The mentor-mentee relationship was relevant here; good mentors could be success factors: *In the beginning, my mentor and I just didn't get along. In the meantime, we understand each other well!* (B6-36, B9-33). A negative or indifferent attitude on the part of the mentor tended to promote a negative attitude in students, even if they had positive prior experience (B8-41, B7-14, B9-45).

#### Assessment of the student representatives

The student representatives unanimously confirmed the differentiated feedback of their fellow students and added relevant hints for improvement. They saw the program in its entirety as a *positive instrument for promoting professional reflectiveness*. Considering that the ability to reflect is very important for the future profession, all students should be convinced of its relevance from the beginning. In this context, they saw the greatest need *in conveying the meaning and purpose* of professional reflection (stronger focus in the introduction). Students should receive regular low-threshold updates with focus topics, as mentors already do. From their point of view, t*he success of the portfolio depended to a large extent on the will, honesty and openness* of the individual students. In their opinion, the decisive factor for the benefits of the SLP was the motivation and trust between the students and the mentors. Overarching aspects that should be considered are: a manageable increase in *study load*, curricular anchor points and integrated assignments to facilitate content connection of reflection, and more creativity and flexibility to develop a personal style of reflection. Overall, they *clearly see the benefits* and support the continued *development* of the SLP. 

## Discussion

This cross-sectional study provides insight into students' attitudes and problem areas in dealing with professional reflection and a reflective portfolio-based mentoring program (SLP) implemented at a German medical school. Responses ranged from full agreement to vehement disagreement or could even change over the course of the study. Depending on the study phase and previous (professional) training, different difficulties became apparent: for students inexperienced with self-reflection, questions toward the meaning and relevance, as well as a lack of skills were particularly problematic, whereas for experienced students study techniques and organizational habits were an issue, and lastly, for both groups, the *hidden curriculum* [8], [27]. The response rate was acceptable and the study population approximately representative for the faculty in Tübingen. The opinion trends of the semester groups confirmed the individual statements. Comparable results were also collected internationally in other study programs including health sciences [22], [28], [29], [30], [31], [32], [33].

In addition to subjective assessments, our study provides supplementary information on the heterogeneity of students, the influence of prior experience, and the role of the hidden curriculum in the German-speaking context. Especially ambivalent students or those showing resistance toward the program hint at the importance of offering a target-group specific design. Notable aspects that influence the perception and impact of the SLP include the students themselves with their individual personality traits, interests, and beliefs. Also included in these aspects are the attitude of the mentors and their relationship to the students, as well as the content and time requirements of the program in general. According to international meta-analyses [2], [8], [19], [22], [34], [35], establishing professional reflection as content in a major requires an all-around understanding of its nature, purpose, and objectives. Lack of knowledge, uncertainties, and misunderstandings make positive access to professional reflection difficult for all involved. Often, students’ own reflective capacities were overestimated, because no distinction was made between the thinking process (deliberation with or without critical evaluation) and the reflective process (balancing deliberation and reflection to develop and evaluate options and plans) [21], [30]. Students who are more accustomed to instructor-driven teaching contexts are more likely to identify self-assessments, solicitation of feedback, reflection, and identification of personal learning needs as a foreign and sometimes even threatening concept [10], [19].

In our study, students with a background in nursing often referred to their already existing reflective competence in terms of their professional self-concept [2], [36], [37]. They saw no need for the SLP for themselves. In their ambivalence, they took too little account of the fact that the development of a new professional identity and accompanying competencies should also be explicitly reflected, as they adapted to modeled behaviors and perceived values of their learning environments (*hidden curriculum*) [38].

Our data shows that the reinforcing role of the *hidden curriculum* operates through three pathways:

1. through the curriculum. Reflective ability and reflective practice [2], [39] are obviously not yet regarded as equivalent to other study related content (above all cognitive subject content), although the foundation for reflective competence has already been introduced in the NKLM.
2. through the faculty. Medical-professionals as positive role models and visible appreciation of reflection by teachers are highly relevant [2], [38]. In their absence, students often adjust their values toward negativity, especially when under the pressure to retain information. Reflection is then, at worst, perceived as a superfluous task that detracts from actual learning. It is imperative to address this question of culture despite resistance [10].
3. through the students. Poor reputation is a risk to a portfolio [19], [24]. Negative attitudes toward it are passed on early among students from one semester to the next. Those who develop their own opinions by being reflective can vastly benefit by taking part in reflective writing and mentor conversations unencumbered and gaining their own experiences.

Also, according to our data, mentor engagement and a trusting mentor-mentee relationship are key elements of student success [2], [10], [24], [40]. They can help identify and work through individual problems, especially for students who show resistance toward the program. Continuous education and guidance, content, and methodological flexibility in combination with reflective writing, self-assessment tools, group discussions, and mentor led conversations can also significantly stimulate student motivation and deepen reflection [16], [41], [42]. 

### Limitations

Data was collected at only one location. The cross-sectional survey is a snapshot of the subjective views of students from all semesters. Intra-individual processes and differential developmental progressions cannot be captured. However, retrospective self-statements by students indicate perceived changes. 

## Implications and future outlook

The high importance of professional reflection makes it necessary to include the teaching of this competence in a medical curriculum. A portfolio-supported mentoring program in a trusting, protected environment can be one method [19] to accompany and strengthen the reflective competence, which can also succeed in other German faculties. Use of an individual, authentic diversity of reflection and target-group specific variability should be integrated [20], [43]. The survey of mentors promises complementary perspectives.

## Funding

The SLP project is part of the joint project MERLIN (Medical Education Research; Phase I: 2012-2016, Phase II: 2017-2020), which was funded by the Federal Ministry of Education and Research of Germany as part of the Teaching Quality Pact (BMBF; reference numbers: 01PL12011A, 01PL17011A).

## Ethical considerations

Ethics committee vote is available (Ethics Committee of the Medical Faculty and University Hospital Tübingen File number 074/2014BO1).

## Authors

### Authors’ contributions

All authors contributed to the conception, design, and discussion of the paper. MLK and JG developed the program and study design. MLK, JG, and JP participated in data collection. SyS, LH, and MLK analyzed and interpreted the data for this paper. MLK, SyS, and LH drafted the manuscript. All authors critically commented on the manuscript, read the completed manuscript, and approved publication of the final version.

#### Notes on contributors

**Sylvia Schrempf** is a pedagogue, surgical assistant, research associate at the Competence Center for University Teaching in Medicine Baden-Wuerttemberg, Faculty of Medicine, University of Tübingen, Germany, and is active in interprofessional adult education.

**Lene Herrigel,** M.A. is a health scientist and research associate at the Competence Center for University Teaching in Medicine Baden-Wuerttemberg, Faculty of Medicine, University of Tübingen, Germany.

**Justus Pohlmann**, cand. med. Is a student of human medicine at the Faculty of Medicine, University of Tübingen and has actively supported the SLP from the beginning as a student representative.

**Jan Griewatz**, M.A. is a pedagogue, research associate and deputy head of the Competence Center for University Teaching in Medicine Baden-Wuerttemberg, Faculty of Medicine, University of Tübingen, Germany.

**Dr. med. Maria Lammerding-Köppel**, MD, is an anatomist with a Master’s degree in Medical Education by Bern University, Switzerland. She was director of the Competence Center for University Teaching in Medicine Baden-Wuerttemberg, Faculty of Medicine, University of Tübingen, Germany from 2001 to 2020.

## Acknowledgement

The authors would like to express their sincere thanks to Tanja Rieß and Maria Farquharson for their valuable preliminary work and support, and to Amir Yousef for his assistance with data analysis. In addition, a heartfelt thank you goes to all students and semester speakers for their extensive comments and constructive suggestions for improvement. We would also like to thank the employees of the Dean of Students Office for their dedicated organizational support.

## Competing interests

The authors declare that they have no competing interests. 

## Figures and Tables

**Table 1:**
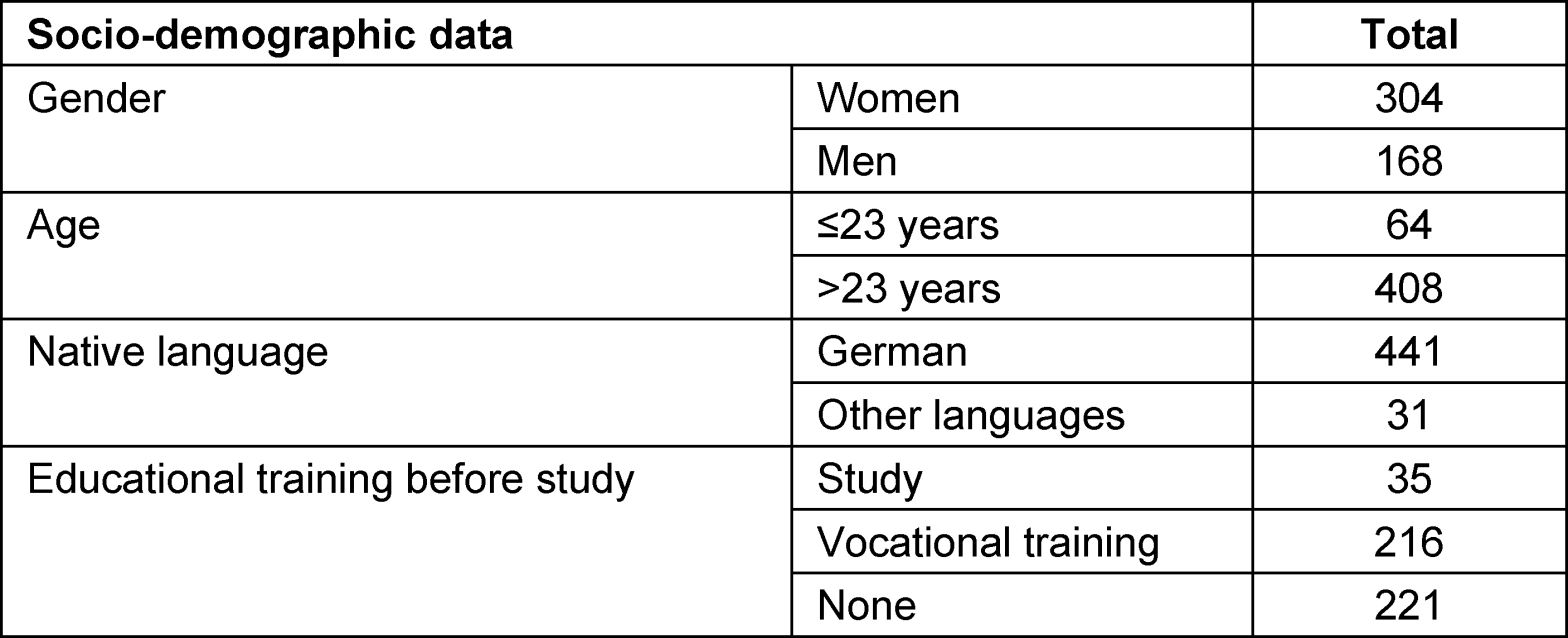
Socio-demographic data of the study participants.

**Table 2:**
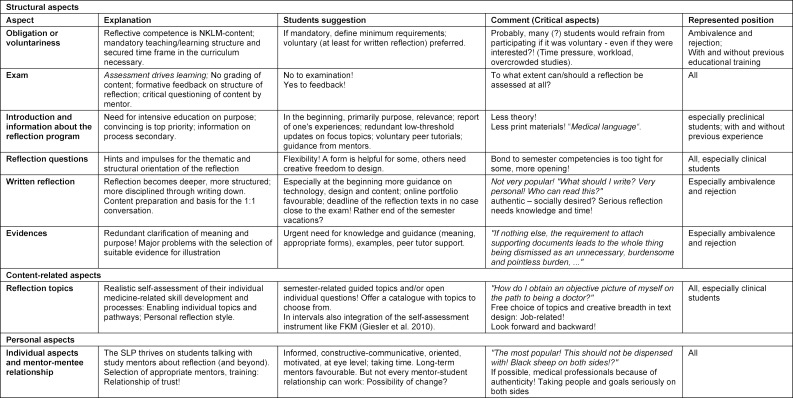
Student feedback on organizational aspects and methodological elements of the SLP. The assessments of the students and their representatives yield a variety of suggestions and hints that can support learner-centered implementation.

**Table 3:**
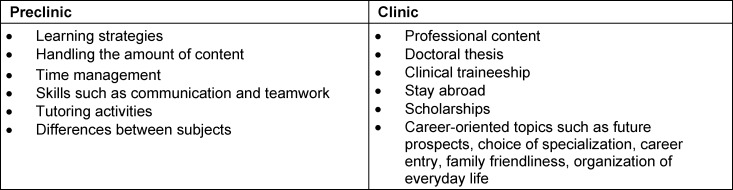
Suggested topics for reflections. Differences in thematic interests between preclinical and clinical students.

**Table 4:**
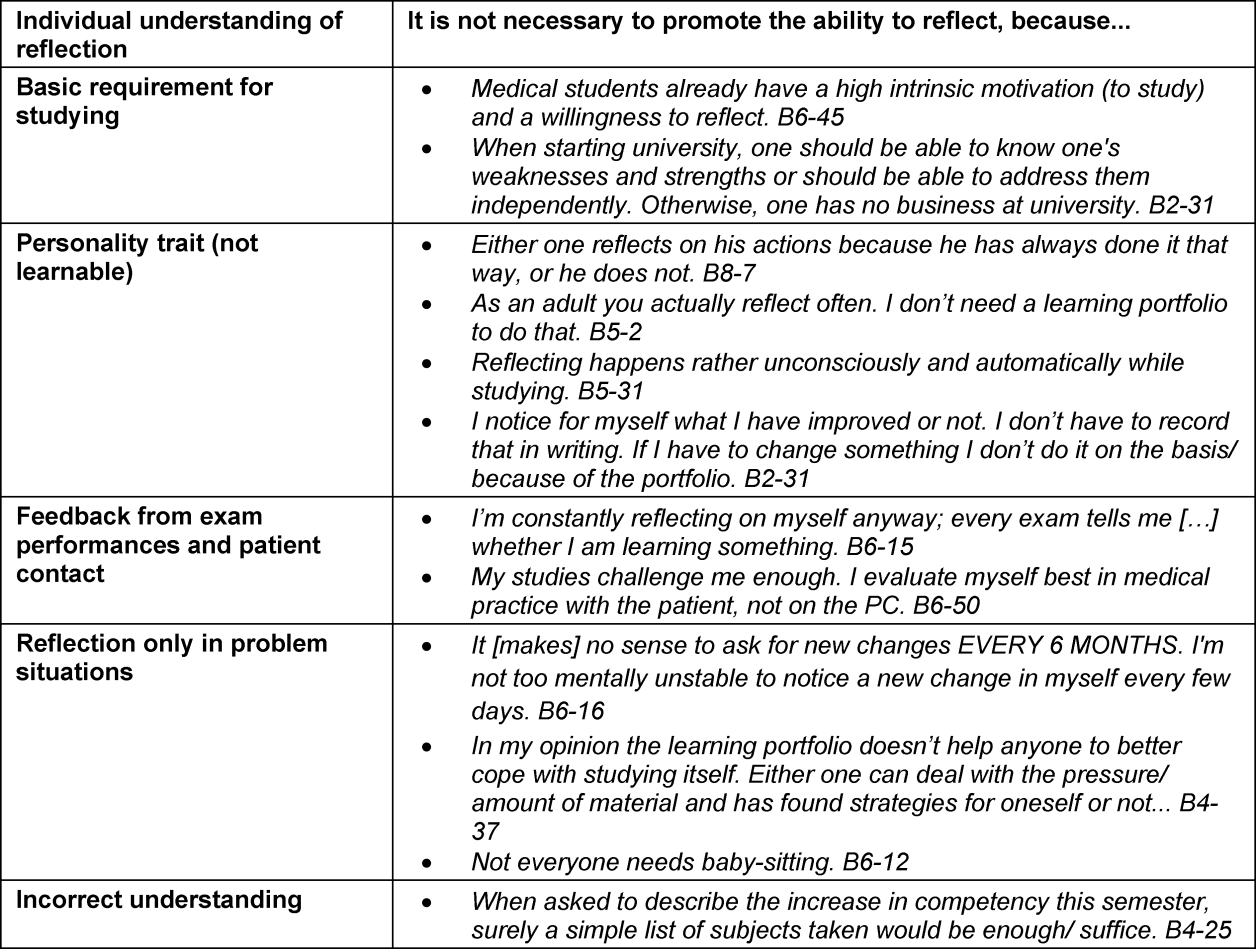
Medical students beliefs explaining their rejection of the SLP (online survey).
